# Epidemiology and reporting characteristics of overviews of reviews of healthcare interventions published 2012–2016: protocol for a systematic review

**DOI:** 10.1186/s13643-017-0468-9

**Published:** 2017-04-07

**Authors:** Dawid Pieper, Michelle Pollock, Ricardo M. Fernandes, Roland Brian Büchter, Lisa Hartling

**Affiliations:** 1 Institute for Research in Operative Medicine, Witten/Herdecke University, Ostmerheimer Str. 200, Building 38, 51109 Cologne, Germany; 2 Alberta Research Centre for Health Evidence, Department of Pediatrics, University of Alberta, Edmonton, Canada; 3 Clinical Pharmacology Unit, Instituto de Medicina Molecular, University of Lisbon, Lisbon, Portugal; 4 Department of Pediatrics, Santa Maria Hospital, Lisbon, Portugal; 5 Institute for Quality and Efficiency in Health Care (IQWiG), Cologne, Germany

**Keywords:** Overview of reviews, Umbrella review, Evidence synthesis, Knowledge synthesis, Evidence-based medicine, Review methods, Systematic reviews

## Abstract

**Background:**

Overviews of systematic reviews (overviews) attempt to systematically retrieve and summarize the results of multiple systematic reviews (SRs) for a given condition or public health problem. Two prior descriptive analyses of overviews found substantial variation in the methodological approaches used in overviews, and deficiencies in reporting of key methodological steps. Since then, new methods have been developed so it is timely to update the prior descriptive analyses. The objectives are to: (1) investigate the epidemiological, descriptive, and reporting characteristics of a random sample of 100 overviews published from 2012 to 2016 and (2) compare these recently published overviews (2012–2016) to those published prior to 2012 (based on the prior descriptive analyses).

**Methods:**

Medline, EMBASE, and CDSR will be searched for overviews published 2012–2016, using a validated search filter for overviews. Only overviews written in English will be included. All titles and abstracts will be screened by one review author; those deemed not relevant will be verified by a second person for exclusion. Full-texts will be assessed for inclusion by two reviewers independently. Of those deemed relevant, a random sample of 100 overviews will be selected for inclusion. Data extraction will be either performed by one reviewer with verification by a second reviewer or by one reviewer only depending on the complexity of the item. Discrepancies at any stage will be resolved by consensus or consulting a third person. Data will be extracted on the epidemiological, descriptive, and reporting characteristics of each overview. Data will be analyzed descriptively. When data are available for both time points (up to 2011 vs. 2012–2016), we will compare characteristics by calculating risk ratios or applying the Mann-Whitney test.

**Discussion:**

Overviews are becoming increasingly valuable evidence syntheses, and the number of published overviews is increasing. However, former analyses found limitations in the conduct and reporting of overviews. This update of a recent sample of overviews will inform whether this has changed, while also identifying areas for further improvement.

**Systematic review registration:**

The review will not be registered in PROSPERO as it does not meet the eligibility criterion of dealing with health-related outcomes.

## Introduction

### Background

Overviews of systematic reviews (overviews) attempt to systematically retrieve and summarize the results of multiple systematic reviews (SRs) for a given condition or public health problem [1]. The number of published overviews has increased steadily in recent years [2, 3]. The Cochrane Collaboration produces overviews, and the number of published protocols and completed overviews in the Cochrane Database of Systematic Reviews (CDSR) has risen to 28 and 26, respectively, at the time of writing (16 December 2016). A preliminary search (conducted on 16 December 2016) in Medline searching *“*review of reviews*”[ti] OR “*overview of reviews*”[ti]* resulted in 92 hits when the search was restricted to the last 5 years, compared to 65 hits before that time range.

Two prior descriptive analyses of overviews found substantial variation in the methodological approaches used in overviews, and deficiencies in reporting of key methodological steps [2, 3]. In both analyses, the most recent fully searched year was 2011. Thus, their findings might no longer be up-to-date.

Since these two descriptive analyses were published, new methods for conducting overviews have been developed or tested, such as methods for dealing with multiple SRs published on the same topic area [4] and methods for displaying outcome data in overviews [5]. Additionally, a new tool to assess the risk of bias in SRs called ROBIS has been published [6]. Reporting quality of SRs has also improved [7–10], and since many reporting items are similar for SRs and overviews an increase in the reporting, quality of overviews might also be expected. Furthermore, a scoping review summarizing existing guidance for conducting overviews of healthcare interventions was recently published [11], and the chapter on overviews in the Cochrane Handbook for Systematic Reviews of Interventions [1] is currently being updated. Both documents provide new guidance on how to conduct overviews, and it can be expected that both guidance documents will be used by authors publishing overviews both in and outside of the CDSR (“Cochrane overviews” and “non-Cochrane overviews”, respectively). This is likely to have an impact on the conduct and reporting of future overviews.

We are not aware of any other analyses describing the characteristics of a recent sample of overviews. Therefore, we considered it timely to update the two prior descriptive analyses conducted by Hartling et al. [2] and Pieper et al. [3].

### Objectives

Our objectives are to: (1) investigate the epidemiological, descriptive, and reporting characteristics of a random sample of overviews published from 2012 to 2016 and (2) compare these recently published overviews (2012–2016) to those published prior to 2012 and previously described [1, 2].

## Methods

### Eligibility criteria

Studies will be selected according to the criteria outlined below.

#### Study designs

To be included in this study, an overview needs to meet the following criteria (modified from [11]):

1. Contain a clearly formulated objective designed to answer a specific research question about a healthcare intervention.
2. Search for and include only SRs (with or without meta-analyses). Note that supplementary searches for primary studies may also be conducted to overcome a deficiency in the SRs identified (e.g., to update an outdated SR, to search for evidence on an important topic not covered in any included SR).
3. Use explicit and reproducible methods to identify relevant SRs that meet their inclusion criteria (e.g., systematic literature search, screening, inclusion).
4. Collect, analyze, and present descriptive and outcome data from the included SRs.

Protocols will be excluded. In cases where updates were published, we will use the most recent version. Overviews embedded within larger health technology assessments or guidelines will be excluded.

#### Language

We will include overviews written in English due to resource constraints.

#### Publication date

We will include overviews published between January 2012 and December 2016 (either printed or electronic).

### Information sources and search strategy

We will search Medline, EMBASE, and the CDSR. Medline via OVID will be searched using a validated search filter for overviews [12]. The same search filter will be adapted to EMBASE via OVID. CDSR via Cochrane Library will be searched using the term “overview” restricted to the title, abstract, and keywords. The search strategy will be limited to the publication years 2012–2016. Searches for gray literature will not be performed as we are interested in published overviews.

### Data management

The search results will be uploaded and managed using Microsoft Excel.

### Selection process

All titles and abstracts will be screened by one review author; those deemed not relevant will be verified by a second person for exclusion (liberal acceleration). Full-texts of all potentially relevant articles and those without an available abstract will be assessed for inclusion by two reviewers independently. Discrepancies will be resolved by consensus or consulting a third person. We will record the reasons for exclusion and report the study selection process using the PRISMA flow diagram [13]. An a priori decision was made to select a random sample of 100 overviews for inclusion in this methods study. Overviews will be selected from the set of screened eligible overviews using the RAND function in Microsoft Excel.

### Data collection process

Prior to data extraction, a data extraction form will be created and tested by the reviewers using a set of five of the 100 included overviews. The extraction form will be modified based on feedback from the reviewers to improve its usability and comprehensibility. To ensure consistency across reviewers, we will conduct calibration exercises before starting data extraction. Data extraction will begin when substantial agreement has been achieved (kappa statistic ≥0.60) [14]. Data extraction will be performed by one reviewer and verified by a second for quality assurance. This will be applied to all items deemed to be difficult in their content (e.g., due to their subjective judgment) among the reviewers after having extracted data on the first five overviews mentioned above. All discrepancies between reviewers will be resolved via consensus or involvement of a third person. For all remaining items, single data extraction will be performed.

### Data items

The data extraction items will be based on those reported in Hartling et al. [2] and Pieper et al. [3] to allow for comparisons. In particular, we will collect data on: journal name, journal type (general vs. specialty), journal impact factor in the year of publication, year of publication, number of authors, country of corresponding author, registration (e.g., PROSPERO), protocol availability (as mentioned or referenced by the authors), topic area (based on International Classification of Diseases-10 (ICD-10)), type of intervention examined (pharmacological, non-pharmacological, both), inclusion criteria clearly stated, primary outcomes stated, number of databases searched, search restrictions (language, date, publication status), selection methods, number of included studies (SRs and primary studies), characteristics of included SRs, data extraction methods, quality and risk of bias assessment of individual studies, quality and risk of bias assessment of systematic reviews, strategies to deal with discordant reviews, update strategies (e.g., searching for primary studies published after the most recent review), strategies for dealing with overlapping reviews, grading of evidence, synthesis, analysis (qualitative vs. quantitative), presentation of results (e.g., harvest plots, vote counting, conceptual frameworks), publication bias, source(s) of funding (Cochrane, academic, government, non-profit organization, industry, other, no funding), and conflicts/declaration of interest.

### Quality assessment

The overall quality of the included overviews will not be assessed, as there is currently no validated tool available to assess the methodological quality of overviews. Further, our data extraction elements listed above include features of methodological (and reporting) quality.

### Data synthesis

Descriptive analyses will be performed using Microsoft Excel software. The analysis will be descriptive, with data summarized as frequencies for categorical items or medians and interquartile ranges for continuous items.

When data are available for both time points, we will also compare the epidemiological, descriptive, and reporting characteristics of the overviews in our sample to the overviews published up to 2011 (contained in the descriptive analyses by Hartling et al. [2] and Pieper et al. [3]). For categorical items, we will calculate the risk ratio, with 95% confidence intervals. The risk ratio is more intuitively interpretable than the odds ratio [15]. For continuous items, we will apply the Mann-Whitney test to compare medians [16]. These analyses will be performed using SPSS Statistics v.21.0 (IBM Corp., Armonk, NY, USA).

Prior to this, we will merge both data sets from Hartling et al. [2] and Pieper et al. [3]. The merged data set will be checked to ensure that all included overviews meet the same inclusion criteria outlined in this protocol. The data for overviews included in both data sets will be checked for consistency. Both steps will be performed by one reviewer and verified by a second reviewer. Any disagreements will be resolved via discussion.

We have not planned any subgroup analyses a priori. However, we may decide to perform post hoc subgroup analyses (e.g., overviews with versus without a protocol) given appropriate numbers per group. They will be determined in consultation with a statistician.

## Discussion

Overviews have become valuable evidence syntheses in the last few years. This is supported by their increasing numbers. Despite the growing interest in overviews, former descriptive analyses found substantial variation in their methodological approaches and reporting quality, thus potentially limiting the credibility and confidence that clinical and policy decision-makers can place in the results and conclusions of overviews. However, both credibility and confidence are necessary for overviews to serve as a useful tool for decision-makers. This update will describe the epidemiology and reporting characteristics of a sample of recently published overviews, while also identifying areas for further improvement in their conduct and reporting. Finally, this work will assist with the development and refinement of guidance for the conduct and reporting of overviews.

## Presenting and reporting the results

This protocol adheres to the *Preferred Reporting Items for Systematic Review and Meta-Analysis-Protocols* (PRISMA-P) [17]. As PRISMA-P aims to guide the development of protocols for SRs evaluating therapeutic efficacy, we deviated from the original checklist by omitting items (e.g., outcomes and prioritization) due to the methodological focus of our planned SR.

## Abbreviations

CDSR: Cochrane Database of Systematic Reviews
ICD-10: International Classification of Diseases-10
PRISMA-P: Preferred Reporting Items for Systematic Review and Meta-Analysis-Protocols
SR: Systematic review
